# Editorial: Host cellular responses to viruses

**DOI:** 10.3389/fmicb.2023.1110197

**Published:** 2023-01-10

**Authors:** Yongqun He, Yongqing Li, Yongchang Cao, Jue Liu

**Affiliations:** ^1^Unit for Laboratory Animal Medicine, Department of Microbiology and Immunology, Center of Computational Medicine and Bioinformatics, University of Michigan Medical School, Ann Arbor, MI, United States; ^2^Research Center for Infectious Diseases in Livestock and Poultry, Beijing Academy of Agriculture and Forestry Sciences, Beijing, China; ^3^State Key Laboratory of Biocontrol, Life Sciences School, Sun Yat-sen University, Guangzhou, China; ^4^College of Veterinary Medicine, Yangzhou University, Yangzhou, China

**Keywords:** virus infection, innate immune response, inflammatory signals, miRNA, host cellular protein networks, pathogenesis

Viruses are intracellular obligate pathogens that rely purely on the host cell for replication. The first step in initiating effective viral infection is to break through the cytomembrane to enter the cell. Next, several adaptor proteins contribute to clathrin-mediated endocytosis of virus entry. After entering the cell, viruses are ultimately dependent on the host cell for their replication *via* altering cellular signal transduction pathways, including IRF3/IRF7, mitogen-activated protein kinases, NF-kappaB, programmed cell death, autophagy, RIG-1-MAVS and cGAS-STING DNA sensing signals, and inflammasome activation signals. Research data documented on the involvement of various cellular proteins or molecules in the replication of viruses have provided essential insights into understanding the viral pathogenesis and have led to the development of antiviral therapeutics. This Research Topic comprises three review articles and 19 research articles from 79 authors, covering many aspects of host cellular responses to DNA and RNA viruses.

The two in-depth reviews by Zhang X.-Z. et al. and Zhou Y. et al. provide readers an up-to-date knowledge of tripartite motif-containing proteins (TRIMs) and Hedgehog (HH) signaling that are targeted by viruses to modulate viral and pathogenesis. Zhang X.-Z. et al. described the regulating functions of TRIMs in host immune signalings and discuss the state-of-the-art research on immune evasion of viruses by targeting TRIMs. Zhou Y. et al. summarized recent advances in functional interaction between HH signaling and viral infections and related diseases. Demonstration of virus-TRIM/HH signaling interactions may provide potential molecular targets for the therapy, or even prevention, of related viral diseases. The third review by Zhang K. et al. reviewed the most recent advances on porcine enteric coronaviruses, including porcine epidemic diarrhea virus, porcine deltacoronavirus, and transmissible gastroenteritis coronavirus, focusing on the molecular mechanisms by which viral components downregulate interferon-modulated innate immune responses, aiming to provide new targets and methods to control and eliminate porcine enteric coronaviruses.

Coxsackievirus B type 3 (CVB3), a member of Enterovirus genus within Picornaviridae, is associated with myocarditis and dilated cardiomyopathy. Qin Y. et al. reported that hsa_circ_0076631 (circ_0076631) significantly enhanced CVB3 replication via modulating viral translation by sponging miR-214-3p, which targeted the CVB3 3D-coding region. Knocking down circ-0076631 resulted in a suppression of CVB3 replication. This study could provide a theoretical basis for clinical treatment of CVB-induced cardiomyopathy.

Bovine herpesvirus 1 (BoHV-1), an alphaherpesvirus, causes infectious rhinotracheitis and pustular vulvovaginitis in cattle. Jiang et al. found that BoIFN-γ pretreatment can significantly inhibit BoHV-1 replication in cultured MDBK cells as evidenced by regulating expression of host protein linked to cellular metabolism and innate immune response, including upregulating ISG transcriptions and expression of IRF1 and GBP5, promoting expression of cellular components participated in complement activation and coagulation cascades as well as antigen presentation and processing, and alleviating metabolism disorder and DNA damage. These findings provide important clues to developing prophylactic measures for prevention and control of BoHV-1 infection.

Severe infection of influenza A virus (IAV) results in overwhelming inflammatory responses, leading to lung injury and high mortality. Liu M. et al. revealed that oral administration of Cangma Huadu (CMHD) granules, a preparation of traditional Chinese medicine, can reduce virus load, inflammatory responses, oxidative stress, and apoptosis in IAV PR8-infected mice. In addition, CMHD granules showed pronounced effects on modulating the diversity and composition of gut microbiota after IAV infection as evidenced by an enhanced abundance of Bacteroides, Bifidobacterium, and Faecalibaculum in PR8-infected mice. This study demonstrated a novel and effective TCM compound, CMHD granules, can be used for the treatment of lethal influenza.

Infection of porcine epidemic diarrhea virus (PEDV) variant has resulted in severe economic losses to the pig industry worldwide. Guo, Yu, et al. found that PEDV infection did not mediate SGs formation in most cultured Vero cells and silencing G3BP1, a stress granules (SGs) marker protein, significantly promoted PEDV replication. Further research showed that PEDV might disrupt SGs formation *via* degrading G3BP1 dependent on activity of viral papain-like proteases. These results illuminated the molecular events participated in the formation of PEDV-induced SGs.

Zhao et al. constructed a novel recombinant bovine herpesvirus type I (BHV-1-ΔgE-G) which expressed rabies virus glycoprotein (RABV G) replaced by its own gE glycoprotein (gE) using CRISPR-Cas9 and homologous recombination technology. Immunization with BHV-1-ΔgE-G can induce a protection against lethal challenge infection in mice. Long-term and protection of RABV-specific neutralizing antibody were detected in mice and cattle. This study demonstrated that the BHV-1 vector-based RABV vaccine can serve as a potential candidate vaccine for cattle.

Infection of Japanese encephalitis virus (JEV) in brain microvascular endothelial cells (BMECs) is considered to be a key step to bring about viral encephalitis. Zhang Y.-G. et al. validated the specific role of activated epidermal growth factor receptor (EGFR) on JEV propagation in human hBMECs. JEV infection induced the phosphorylation of EGFR and its downstream signals. Using specific inhibitors or knocking-out of EGFR showed that EGFR assists JEV virions production by negatively modulating the antiviral interferon response, but does not involve viral attachment or entry. These results revealed the mechanism of how JEV exploits EGFR signaling to promote viral replication, thereby providing a potential target for therapy for infection of JEV.

The transposition of long interspersed element 1 (LINE-1) can result in genetic instability/diseases. Zhang Z. et al. reported that CCHC-type zinc-finger protein ZCCHC3 inhibited LINE-1 retrotransposition via its zinc-finger domain and diminished the LINE-1 RNA level. These results of contribution of ZCCHC3 to LINE-1 replication help avoid the adverse effects of LINE-1 transposition on the host genomic structure and function.

Latorre and Geller identified various members of host cellular protein folding networks involving in respiratory syncytial virus (RSV) replication. The decreased number of chaperones and co-chaperones can facilitate the unmasking of certain chaperone subnetworks essential for critical steps of the RSV life cycle. This study improved our understanding of this host-pathogen interface and revealing new potential targets for therapeutic intervention.

Fiacchini et al. found that the COVID-19 tracheal samples showed a significant alteration of two sets of gene expression related to activation of the proinflammatory response and inhibition of the estrogen response as compared to the non-COVID-19 controls. The altered inflammatory response in the COVID-19 patients could be a possible explanation of the enhancing number of laryngotracheal complications.

Zhou H. et al. observed that the expression of CCN1 was enhanced by PEDV infection and found that porcine epidemic diarrhea virus (PEDV) infection promoted the phosphorylation of CREB and c-Jun in the nucleus through the PKA and p38 pathways, and increased the production of CCN1. Further research showed that the overexpression of CCN1 decreases PEDV replication and promotes p53-dependent apoptosis as well as the knockdown of CCN1 enhanced PEDV proliferation and suppresses p53-dependent apoptosis in Marc-145 cells. This study provides important understanding for the molecular mechanism of PEDV pathogenesis.

Endoplasmic reticulum aminopeptidase 1 (ERAP1) is an important processing enzyme of antigenic peptides which are presented to major histocompatibility complex class I (MHC-I) molecules. Trimming of epitope repertoire dependent on ERAP1 correlates to efficacy of CD8+ T-cell responses in some viral diseases. Liu H. et al. showed that ERAP1 trims HBcAg to generate 9-mers LLDTASALY peptide for binding to HLA-C^*^04:01 of HepG2.2.15 cells, promoting activation of CD8+ T cells. These findings revealed a previously unknown HBV viral antigen peptides presentation–based immune mechanism that targets a critical step in the MHC-I antigen-processing pathway.

Singapore grouper iridovirus (SGIV), a member within the Iridoviridae family, is a major marine cultured fish causative agent worldwide. Guo, Zhang, et al. determined the expression levels of critical enzymes of glucose metabolism during SGIV infection and demonstrated that glycolysis might be involved in infection of SGIV using specific pharmacological inhibitors and siRNA technology. They also clarified the role of mTOR in glycolysis induced by SGIV. These results provided novel insights into the mechanism in which SGIV infection affected host cell glycolysis and contributed to deep understanding of iridovirus pathogenesis.

Yan et al. investigated the kinetics of neutralizing antibody and T cell response to SARS-CoV-2 in COVID-19 patients and found that most patients had developed and maintained a long term effective neutralizing antibodies and a specific T cell responses to SARS-CoV-2 in at least one and half years after the onset of illness during the study period, even in mild individuals. A positive correlation was seen between SARS-CoV-2-specific neutralizing antibody levels and T-cell responses. This study indicated that effective long-term herd immunity could be achieved through global vaccination.

Lee et al. identified a novel anti-HBV signaling pathway as evidenced by that hepatocyte growth factor (HGF)-induced ACK1 (activated cdc42-associated kinase 1) inhibited HBV gene expression and replication by regulation of Erk1/2-HNF4α signaling cascade. Notably, HGF reduces the HBV replication and cccDNA in HBV-infected cells and ACK1 is involved in anti-HBV activity of HGF. These findings provide a new signaling for anti-HBV activity of HGF during the regeneration of damaged liver tissue by persistent infection of HBV.

Liu P. et al. demonstrated that meclizine showed a significant inhibition against Pseudorabies virus (PRV) *via* interfering with virus entry, cell-to-cell spread, and release in cultured cells. Further animal experiments demonstrated that meclizine decreased the severity of clinical signs and the viral burdens in tissues and delayed the death of mice after PRV challenge. These results lay a foundation for the development of PRV antiviral drugs, thereby providing a new perspective to the study of antiviral mechanism.

Sufficient presence of the hepatitis B virus (HBV) covalently closed circular DNA (cccDNA) is considered to be the failure of antiviral therapies. Qin Y.-P. et al. identified KAT2A as a crucial host factor in HBV transcription and replication by screening a series of succinyltransferases and desuccinylases. KAT2A was shown to affect cccDNA transcriptional activity but not cccDNA production in HBV-infected cells and mouse models. Silencing KAT2A could restrict cccDNA transcription activity by decreasing the level of H3K79succ on cccDNA minichromosomes. These findings demonstrated that KAT2A enhances HBV transcription and replication *via* epigenetic machinery, thereby providing new insight into the therapy of hepatitis B virus infection.

Cyprinid herpesvirus 2 (CyHV-2) has resulted in severe economic loss to the Crucian carp breeding industry. Lu et al. showed that antioxidant-related gene expressions were upregulated in cultured CyHV-2-infected Ryuf-2 cells and found that antioxidants can effectively inhibit the replication of CyHV-2 through reduced reactive oxygen species production.

Chen et al. identified a lnc-LTR5B induced by endogenous retroviral LTR. This lnc-LTR5B participates in regulating translocation of BiP to the cell surface, and the avian leukosis virus subgroup J can exploit such regulatory machinery for completing can be exploited by to complete its life cycle and propagation. This study provides a virus-based lncRNA-modulated mechanism that facilitates to develop new antiviral strategies.

Ma and Niu analyzed the metabolic profiles of hepatocellular carcinoma cell line when infected with fowl adenovirus serotype 4 (FAdV-4), which is the major pathogen of hydropericardium syndrome severely damaging to poultry farming. FAdV-4 was shown to modulate glycolysis, tricarboxylic acid cycle, and metabolism of alanine, aspartate, purines, pyrimidines, glutamate, and sugar moieties in the cultured cells. These findings provide new insights into developing prophylactic measures for prevention and control of FAdV-4 infection.

Selected articles in this Research Topic range from our basic understanding of host cellular responses to virus infections to potential approaches to prevention and therapy for viral diseases. The editors of this topic sincerely thank all authors and reviewers for their contributions.

## Author contributions

All authors listed have made a substantial, direct, and intellectual contribution to the work and approved it for publication.

